# Electrical nerve stimulation for sensory-neural pathway reconstruction in upper-limb amputees

**DOI:** 10.3389/fnins.2023.1114962

**Published:** 2023-02-09

**Authors:** Yingying Wang, Hongyu Liu, Peiying Zeng, Lingchao Ji, Yaqi Zhou, Liufang Zhou, Yuan Tao

**Affiliations:** ^1^Department of Otolaryngology, Peking University Shenzhen Hospital, Shenzhen, China; ^2^CAS Key Laboratory of Human-Machine Intelligence-Synergy Systems, Shenzhen Institute of Advanced Technology, Chinese Academy of Sciences, Shenzhen, China; ^3^Department of Rheumatology and Immunology, Peking University Shenzhen Hospital, Shenzhen, China

**Keywords:** transcutaneous electrical nerve stimulation, neural pathway, natural sensory transmission, physical stressor, brain activity, phantom hand map, electroencephalogram (EEG)

## Abstract

**Introduction:**

The loss of the neural sensory function pathways between the stump limbs and the brain greatly impacts the rehabilitation of limb function and the daily lives of amputees. Non-invasive physical stressors, such as mechanical pressure and transcutaneous electrical nerve stimulation (TENS), could be potential solutions for recovering somatic sensations in amputees. Previous studies have shown that stimulating the residual or regenerated nerves in the stumps of some amputees can produce phantom hand sensations. However, the results are inconclusive due to unstable physiological responses caused by inaccurate stimulus parameters and positions.

**Methods:**

In this study, we developed an optimal TENS strategy by mapping the distribution of the nerves in the stump skin that elicitsphantom sensations known as a “phantom hand map.” We evaluated the effectiveness and stability of the confirmed stimulus configuration in a long-term experiment using single- and multi-stimulus paradigms. Additionally, we evaluated the evoked sensations by recording electroencephalograms (EEG) and analyzing brain activities.

**Results:**

The results demonstrated that various types of intuitive sensations for amputees could be stably induced by adjusting TENS frequencies, particularly at 5 and 50 Hz. At these frequencies, 100% stability of sensory types was achieved when the stimuli were applied to two specific locations on the stump skin. Furthermore, at these locations, the stability of sensory positions was 100% across different days. Moreover, the evoked sensations were objectively supported by specific patterns of event-related potentials in brain responses.

**Discussion:**

This study provides an effective method for developing and evaluating physical stressor stimulus strategies, which could play an important role in the somatosensory rehabilitation of amputees and other patients suffering from somatomotor sensory dysfunction. The paradigm developed in this study can provide effective guidelines for stimulus parameters in physical and electrical nerve stimulation treatments for a variety of symptoms related to neurological disorders.

## 1. Introduction

Existing commercial prosthetic hands still rely on vision or acoustic feedback, reducing the acceptability of these devices and negatively affecting amputees' confidence in using them (Makin et al., 2017). An ideal prosthetic hand should have the capability to provide perceptions similar to those of an intact limb, including sensation qualities and locations (Raspopovic et al., 2021). Therefore, one of the most significant research areas is somatotopic and homologous sensory restoration, which mainly relies on developing effective external stimulation methods.

The method of stimulation is crucial for inducing intuitive sensations. Earlier research reported that different types of microelectrodes could be implanted in the elementary somatosensory area of the brain (Flesher et al., 2016, 2017) or the peripheral nerves (Overstreet et al., 2019; Zollo et al., 2019), evoking haptic sensations that are perceived as originating from different locations on the hand. In one study (Granata et al., 2018), TIMEs were implanted in amputees' medians and ulnar nerves, which then evoked sensations in different parts of the phantom hand by adjusting the time intervals between different stimuli channels. Afterward, more stimulus modes on more amputees were tested by comparing different channel combinations, resulting in the induction of complex feelings, including different sensation locations and types (Strauss et al., 2019). However, some limitations prevent the widespread application of invasive electrical stimulation in the sensory feedback of prosthetic devices for amputees, such as battery capacity, the size of the implanted systems, additional medical and healthcare costs after surgery, scarring, and the penetration caused by electrode arrays, which may cause scars that impair signal acquisition (Valle et al., 2018). Therefore, a noninvasive nerve interface is imperative for the restoration of tactile sensations, which includes electro-tactile stimulation based on mechanoreceptors and transcutaneous electrical nerve stimulation (TENS) (Li et al., 2017). More recently, a new (Gu et al., 2021) type of neuroprosthetic hand was reported in the amputee's upper arm, where stimulus electrodes were attached to the skin surface of the transradial amputee's upper arm, and artificial tactile sensors were installed on five fingertips of the prosthetic hand. Each prosthetic finger was mapped to a corresponding stimulus electrode, resulting in the sensations of single and multiple prosthetic fingers. However, the need for intuitive sensory feedback remains unsatisfactory because this approach could not activate the nerves to induce the real sensation of the phantom fingers that are innervated. In contrast, TENS technology can play a valuable role in evoking somatotopically matched sensations (Svensson et al., 2017). Osborn and Henry et al. applied TENS to the median and the ulnar nerves of the upper residual arms, evoking painful and non-painful sensations to recognize objects with different curvatures by quantifying stimulation parameters (Osborn et al., 2018) and realizing selective haptic sensations by using an electrode array (Shin et al., 2018). D'Anna et al. also used the TENS technology to stimulate the median and ulnar nerves to induce sensations of phantom hands in amputees (D'Anna et al., 2017). However, the delivered stimulation activated most of the large-diameter nerve fibers whose receptive fields cover large areas of the phantom hand, making it difficult to distinguish sensations between different fingers or the palm, although they were somatotopically matched.

A review report shows that amputees with a phantom hand map (PHM) on their stumps are more likely to experience spontaneous sensations induced by TENS (Svensson et al., 2017). Notably, Kuiken et al. developed the targeted muscular reinnervation (TMR) technology which offers a new approach to restoring the motor and sensory functions for amputees (Kuiken et al., 2007a, 2009). A previous study (Kuiken et al., 2007b) confirmed that amputees with high levels of amputation had clear PHMs of their lost hands on their chest skin after TMR, and they experienced intuitive sensations in their lost hands and arms when exposed to stimulation, such as mechanical pressure, temperature, or electricity. Additionally, Henrik et al. randomly selected 18 forearm amputees for a tactile illusion stimulation experiment, 12 of whom have a PHM on their stumps (Ehrsson et al., 2009). The results showed that patients with a PHM had better tactile induction performance, and five of the six patients with the strongest sensations had a PHM on their stumps. Furthermore, many TENS-based studies on restoring sensory function have shown that more than 66.7% of the investigated amputee subjects have a PHM on their stumps (Schmalzl et al., 2011; Bjorkman et al., 2012; Antfolk et al., 2013; Chai et al., 2015; Björkman et al., 2016; Hao et al., 2020). They found that different amputees have different PHMs (such as qualities, size, and distribution) due to their amputation reasons, stump lengths, stump conditions, and so on. As a result, the evoked sensations are also different (Schmalzl et al., 2011; Bjorkman et al., 2012; Antfolk et al., 2013; Chai et al., 2015; Björkman et al., 2016; Hao et al., 2020). The more distal the amputation level, the more detailed the representation of fingers in the PHM. Besides, positions with more detailed and clearer PHMs on the amputees' stumps had stronger sensitivity to electrical stimulation, making it easier to evoke and distinguish sensations of different phantom fingers in these positions (Chai et al., 2015). Additionally, regular PHMs also make it easier to position the stimulus electrodes, a vital factor for successful sensation induction. Our previous studies also proved that the phantom fingers' sensations could only be successfully induced when TENS is applied to appropriate PHMs (Wang et al., 2022). However, the mechanism of PHM formation is still unclear, and the two most plausible theories, such as peripheral nerve regeneration of the stump and extensive cerebral reorganization after amputation, are not yet fully understood (Björkman et al., 2016; Strauss et al., 2019). Moreover, a major challenge remains in determining the optimal strategy for identifying effective stimulus positions and parameters for inducing phantom finger sensations. Currently, the effectiveness of TENS in inducing natural intuitive sensations is still insufficient to meet the needs of amputees and in clinical applications. To overcome the above limitations, the main purpose of this study is to provide effective TENS configurations based on the PHM investigation. Thus, subjects with amputations caused by different reasons, such as tumors and trauma, were recruited for the experiment. The distribution of PHM was explored in detail through a long-term follow-up experiment to optimize the stimulation position. Besides, many stimulation parameters, including waveform, amplitude, frequency, and wave width, were tested to confirm the optimal parameter combinations. Furthermore, the stability and consistency of the sensations evoked by TENS with the confirmed parameters for different amputees were tested. Finally, the evoked sensations were evaluated by recording the EEG of one amputee subject. This study would contribute to the development of noninvasive sensory feedback systems that could offer somatotopically matched sensation to prosthetic users and improve the ownership of their prostheses.

## 2. Methods and materials

### 2.1. Subjects

Four male subjects whose left or right forearms were amputated for 7.5 ± 6.5 years due to accidental trauma or disease (with an average age of 27 ± 9, an average height of 175.5 ± 7.5 cm, and an average weight of 77 ± 23 kg) were recruited for the study. Detailed information about subjects, including reasons, timing, and positions of amputation, is listed in Table 1. A preliminary health examination showed that all the subjects were in a good mental state and could participate in the study. The research protocol was approved by the Institutional Review Board of the Shenzhen Institutes of Advanced Technology, Chinese Academy of Sciences (IRB Number: SIAT-IRB-190315-H0325). All the recruited subjects gave their written informed consent and provided permission for the publication of photographs only for scientific and educational purposes.

**Table 1:** Information about subjects.

	**Age (years)**	**Height (cm)**	**Weight (kg)**	**Stump length (cm)**	**Duration of amputation (years)**	**Cause of amputation**	**PHM (yes/no)**
**Sub1**	36	168	68	15	14	Industrial machine	Yes
**Sub2**	26	168	54	14	3		
**Sub3**	18	183	100	25	1	Industrial machine & High-temperature burns	
**Sub4**	26	170	55	14	2	Disease	

### 2.2. Development and optimization of stimulus strategies

#### 2.2.1. Accurate stimulus positions confirmed based on PHM

**Figure 1:**
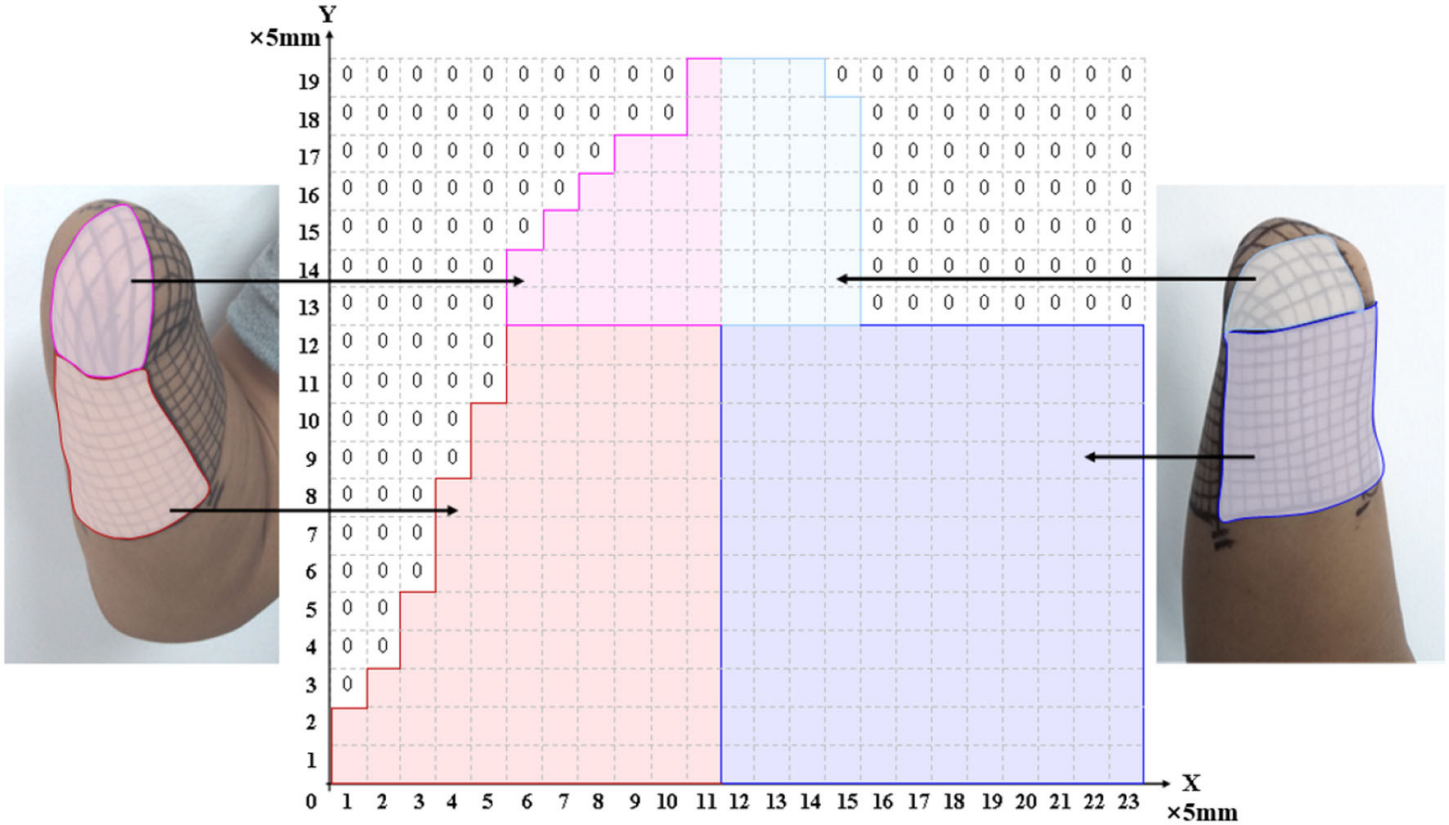
The relationship between the meshed coordinate and the skin region of PHMs.

The PHM distribution on the stump skin was explored using a mesh coordinate method to determine appropriate stimulus positions for evoking stable, somatotopically matched sensations. For *subject 1*, a coordinate with 19 × 23 cells (with an *x-*axis from 0 to 23 and a *y-*axis from 0 to 19) was plotted on his stump skin to map his residual limb in ach cell. It was a 5 mm × 5 mm grid and was considered a single measurement unit, as shown in Figure 1. The relationship between the coordinates and the skin areas that were measured on the residual limb is illustrated in Figure 1. Then, each grid was stimulated with mechanical pressure three times using a small rod (≈ 3 mm in diameter), and the subject's feelings were recorded. Furthermore, the numbers I-VI were used to label the locations of the sensations evoked by stimulating each cell (I for the thumb, II for the index finger, III for the middle finger, IV for the ring finger, V for the little finger, and VI for the palm), based on the subject's reports. This exploration progress was repeated every 3–5 days, and a total of eight times were performed, supporting us in exploring the law of PHM distribution and then determining the appropriate positions for electrical stimulation. Finally, the distribution of PHMs was obtained, including the accurate spot of each phantom finger. The PHM size for each phantom finger was obtained by counting the cells covered by PHMs on that finger. Then, the possible effective stimulus positions, which were the potential candidates for the following TENS tests, were determined.

#### 2.2.2. Selections of stimulus waveforms and parameter ranges

**Figure 2:**
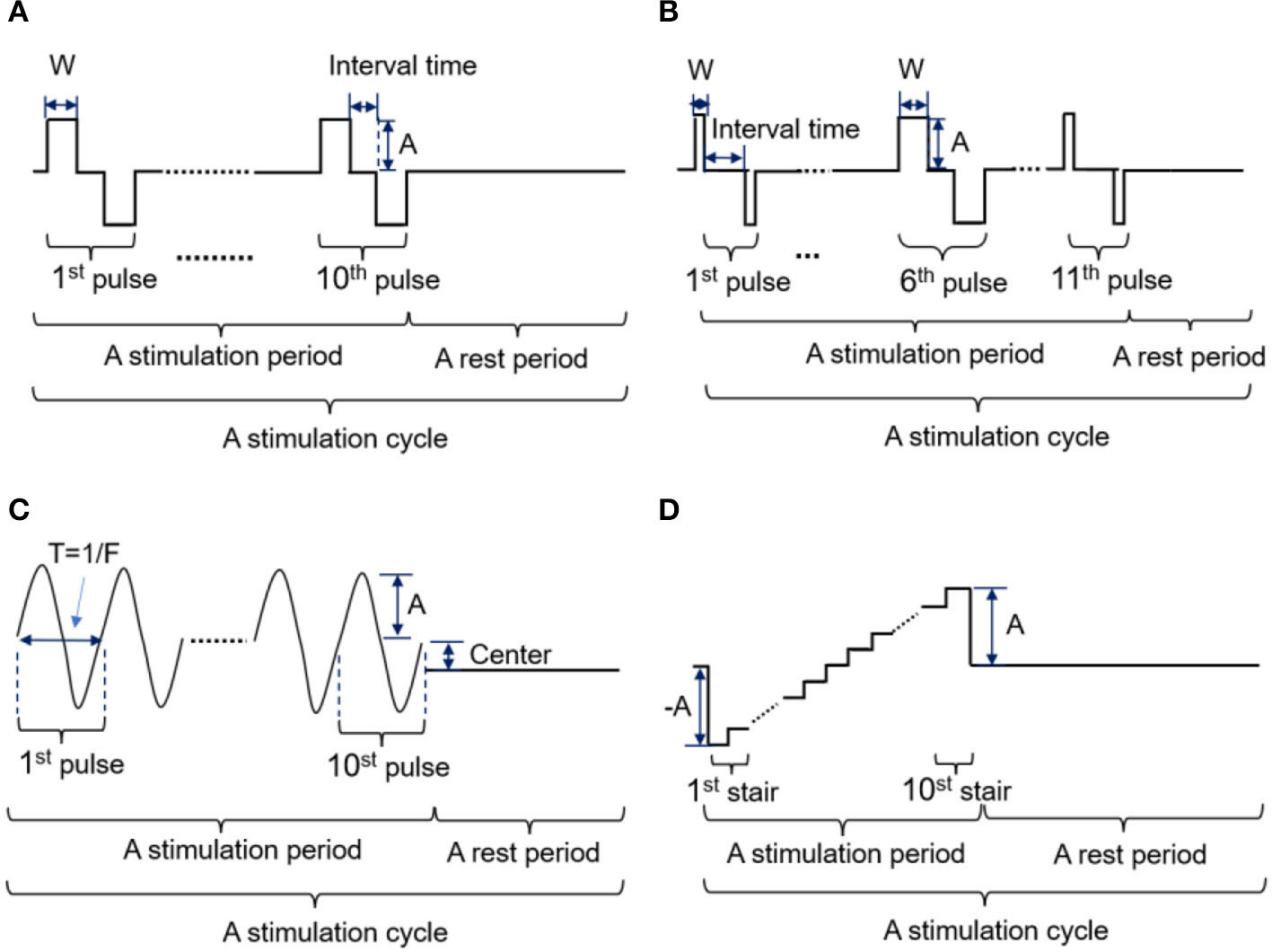
**(A-D)** Four kinds of stimulation waveforms were tested in the selection experiment parameter ranges.

In this study, four kinds of stimulation waveforms were tested using a 1-s test cycle. The first waveform was a bidirectional rectangle with a fixed width (W) of 200 μs, an adjustable frequency (F) of 50 Hz, and an amplitude (A) range of 0.5–5 mA. This waveform included 10 pairs of bidirectional pulses with a 200 μs interval between the positive and negative pulses, as shown in Figure 2A. The second waveform was a bidirectional rectangle with a variable width of 80 to 200 to 80 μs, a frequency (F) of 50 Hz, an amplitude (A) range of 2–5 mA, and 11 pairs of pulses with an interval time of 200 μs, as shown in Figure 2B and a sinusoidal wave consisting of 10 complete, continuous sine pulses with a center of 0.5 mA, a fixed frequency (F) of 50 Hz, and an amplitude range of 2–5 mA, as shown in Figure 2C. The fourth waveform was a stair wave featuring 10 stairs with a maximum amplitude of 0 mA and an amplitude range of 2–5 mA, as shown in Figure 2D. A waveform generator (*CED Micro 1401-4, Digitimer, UK*) was used to set up different types of electrical stimulation sequences by changing the duty ratio and combining pulses of various widths. These stimulation sequences were then output and delivered to the target stimulation site through an isolated constant-current stimulator (*DS5, Digitimer, UK*). The stimulation electrodes were attached to the designated stimulus positions on the stump, as determined by the PHM distribution obtained in section 2.2.1. The subjects' responses (including the presence or absence of sensation and the position, type, intensity, and comfort of any evoked sensation) were recorded while different stimulus currents were applied to the electrodes. This procedure was performed two times, with a 3 day interval between sessions. Finally, the waveform and ranges of parameters of the potentially effective stimulus were recorded for use in subsequent experiments.

#### 2.2.3. Assessments of selected parameters and evoked sensations

Afterward, TENS with different parameters within the selected ranges was applied to the targeted positions, and all kinds of sensations evoked by the TENS were recorded. This part of the experiment was carried out six times with intervals of 3–7 days, with the aim of exploring possible stimulus thresholds and inferring potential relationships between stimulation parameters and evoked sensations. Afterward, the optimal stimulation parameters were confirmed, which were used for a long-term stability test in the next step. In this study, sensation intensity was quantitatively evaluated by assigning it a numerical value on a scale of 0–10 based on the subject's feedback. The “0” indicates that no sensation was evoked by the TENS and felt by the subject, while the “1” and “10” represent the minimum and maximum sensation thresholds, respectively. Concretely, “1” indicates that an extremely slight sense of the induced phantom finger sensation was perceived by the subject (called the “initial feeling”), and subjects should remember that “initial feeling. Afterward, they need to compare the intensity of the current feeling with that of the “initial feeling” and assign a value on a scale of 1–10 based on the relative intensity of the evoked sensation. Additionally, “10” indicates that nociception, like burning and pain sensations, such as obvious tingling, numbness, burning, etc., were felt or the phantom fingers were beginning to shake (called the “terminal feeling”). Furthermore, the stimulation current threshold was found according to the sensory intensity. Here, the threshold refers to the corresponding stimulus parameter when the subject is only beginning to feel the slight sensation of the finger or palm, that is, the parameter when the sensory intensity is marked “1.” In this study, only the amplitude threshold was focused, so the stimulation was carried out with increasing stimulus amplitudes at a frequency of 50 Hz and a wave width of 200 μs.

The stability of the corresponding relationships between the determined stimulus positions, parameters, and the evoked sensations (including sensory positions, types, and different levels of intensity) was tested through a long-term tracking experiment. This part of the test was carried out in two types of electrical stimulation patterns, single stimulation (SS) and multiple stimulations (MS), with intervals of three to seven days, for a total of eight times. The SS pattern only contained one stimulus cycle in one trial for each set of parameters. The MS pattern involved 50 stimulus cycles in one trial with a 6-s interval after each cycle (i.e., 5 min for one trial). After the long-term test experiment, the locations, types, and different levels of intensity of the sensations, which were evoked by the TENS at each determined stimulus position under each selected set of stimulus parameters, were counted, and their respective frequencies in all experiments were calculated. For instance, the frequency of each phantom finger position evoked at one stimulus location was calculated as the ratio of the number of times that the finger position appeared at that stimulus position to the total number of all possible finger positions evoked at that stimulus position in all experiments. This calculation method for the frequency of positions was also used for the frequency of sensation types. In the end, the stable relationships among stimulation positions, parameters, different levels of intensity, and evoked sensations were confirmed, and the stimulus parameters with the best effectiveness in phantom finger sensation induction were determined.

### 2.3. Analysis of evoked sensation-related EEG

While the TENS in the MS pattern acted on the determined stimulus positions, EEG signals were recorded simultaneously to explore neural correlations of evoked sensations. The professional acquisition system (*64-channel Quik-Cap* & *SynAmps 2, NeuroScan, USA*) was used to collect EEG signals; the sample rate was 1,000 Hz, and the impedance on most electrodes was kept within 5 kΩ. Besides, the subject was sitting on a chair in an electromagnetically shielded room without light or acoustics, keeping a fixed posture and attempting to not blink his eye, swallow, or move his head. The EEGLAB toolbox was used to analyze EEG signals in MATLAB. The poor-quality signal epochs that contained clear artifacts like eye movements and electrode slipping were also removed. Besides, abnormal electrodes, which have a too high impedance to collect real EEG signals caused by some accidental pulling, among others, were replaced by using an interpolation method based on their neighboring normal electrodes. To better understand how the brain responds to the TENS, the signals were divided into five segments *via* five passband filters, which, respectively, correspond to five kinds of EEG rhythms, namely, theta (θ: 4–8 Hz), alpha (α: 8–13 Hz), low beta (lβ: 13–21 Hz), high beta (hβ: 21–30 Hz), and gamma (γ: >30 Hz). For the signal segment under each frequency band, the independent component analysis (ICA) methodology was performed to maximize and optimize the reliability and polarity of the event-related potentials (ERPs) extracted from the EEG signals. Then, each component obtained from ICA was analyzed through scalp maps, and the bad component containing too many artifacts was manually removed. Afterward, the individual channel ERPs were calculated, and the channels related to electrical stimulation were selected by setting the threshold according to equation (1), where *L* represents an ERP segment when there is no stimulation carried out on the subject. Channels for those ERP peak values that exceeded the range from th1 to th2 were selected and averaged. Finally, averaged-ERP data for five frequency bands were obtained, and the peak values of the averaged-ERP curve were compared for different evoked sensations.


(1) $$\begin{array}{l} th1\;=\max\left(L\right)+\frac{\max\left(L\right)-\min\left(L\right)}{2},\;\\ \;th2\;=\min\left(L\right)-\frac{\min\left(L\right)-\max\left(L\right)}{2} \end{array}$$

## 3. Results

### 3.1. Distributions of PHMs

**Figure 3:**
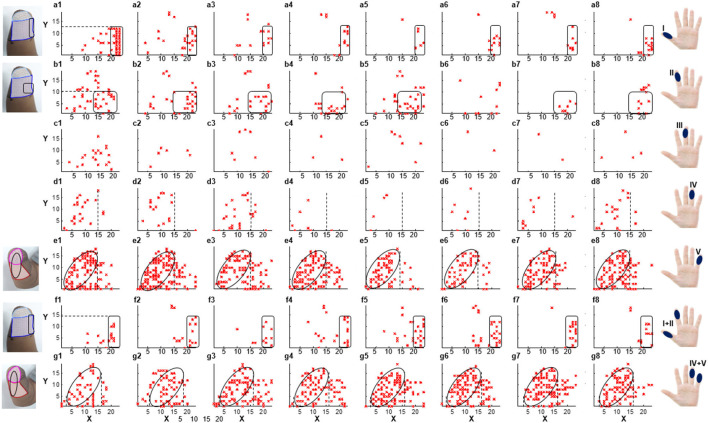
The distribution of PHMs for the thumb (I), index (II), middle (III), ring (IV), and little (V) fingers, as well as the mixture of the thumb and index fingers (I+II), the ring and little fingers (IV+V). Both *X-*axis and *Y-*axis numbers correspond to the respective positions in the previous meshed coordinates.

According to the result of PHM distribution, for subjects 1, 2, and 4, sensations of five phantom fingers can be evoked by mechanical stimulations, so corresponding areas were found within PHMs. However, for *subject 3*, only PHM areas for the thumb, index, and little fingers were identified. Figure 3 shows the coordinates of scatter points that represent areas of PHM for the phantom thumb, index, middle, ring, and little fingers (I, II, III, IV, and V) for *subject 1* during eight repeated experiments. It demonstrates some distribution laws of PHM areas, such as the most PHM for the thumb is concentrated in the region defined by *X-*axis coordinates of 20–23 and *y-*axis coordinates of 0–12 (as shown in Figures 3a1–a8). However, in most of the experiment repetitions, the PHM of the index finger is more concentrated in the region defined by *X-*axis coordinates 15–23 and *Y-*axis coordinates 0–10 than in other regions (as shown in Figures 3b1–b5, b7, b8). Nevertheless, PHM regions for other fingers are relatively dispersed, especially for the middle finger (as shown in Figures 3c1–c8). The PHM of the ring finger is roughly dispersed in areas where the values of the *X-*coordinate are smaller than 15 (as shown in Figures 3d1–d8). Moreover, the PHM of the little finger almost covers the whole axes except for the region where coordinate values are between 2 and 15 on the *X-*axis and < 19 on the *Y-*axis (as shown in Figures 3e1–e8). Besides, it is noticeable that some PHMs with mixed fingers exist in many regions, and each of them contains more than one phantom finger sensation. For instance, the PHM for the mixture of the thumb and the index finger is more intense in areas defined by *X* coordinates 19–23 and Y coordinates 0–15 than in other regions (as shown in Figures 3f1–f8). The PHM for the mixture of the ring and little fingers covers approximately the same region as the PHM for the little finger (as shown in Figures 3g1–g8). In addition, the total number of cells across eight experiments representing PHM areas for each finger was counted to measure the size of PHMs for different kinds of fingers (Supplementary Figure S1). The size of PHM for the little finger was discovered to be the largest (90), followed by a mixture of the ring and little fingers (80). Furthermore, PHMs for the thumb, index, and ring fingers each had the same number of occurrences in the grid (20) as PHMs for three different types of mixed fingers: thumb and index finger (I+II), middle and ring finger (III+IV), and index, ringer, and little finger (II+IV+V), respectively.

### 3.2. Parameters and positions for sensation induction

**Figure 4:**
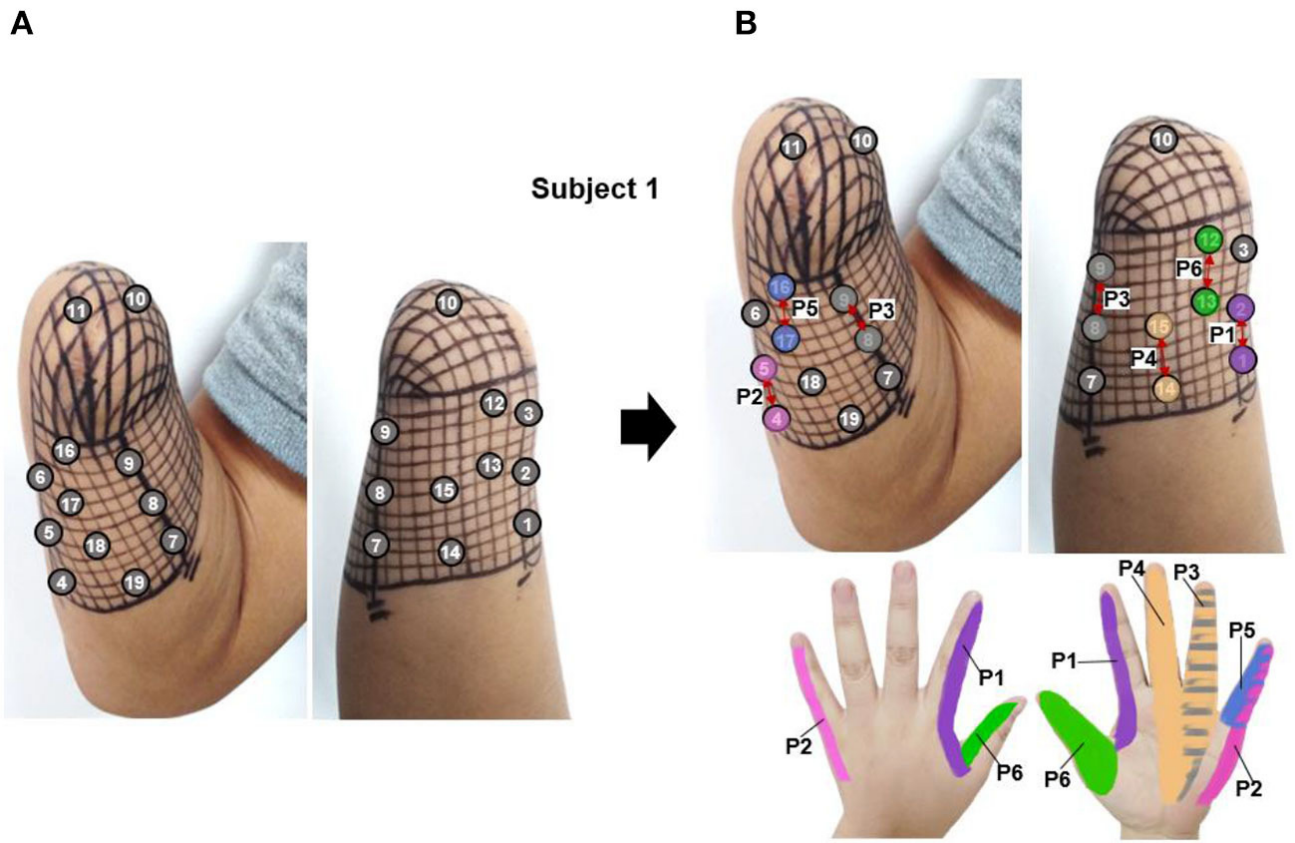
The selection of stimulus locations for subject 1, **(A)** the preliminarily selected nineteen stimulation electrodes based on the PHM distribution for sensation induction *via* TENS, **(B)** the determined six appropriate stimulus positions, which were marked by red arrows (P1–P6).

According to the PHM distribution law obtained in section 3.1, there are some areas where PHMs for the same finger are concentrated. Figure 4A shows the preliminary selection of stimulus positions for *subject 1*. It illustrates that 19 potential PHM areas, each corresponding to a specific phantom hand position, were selected. Then, the stimulation electrode was attached to each selected area, and the sensor*y-*evoked performance of four kinds of stimulus waveforms was tested by applying TENS to each pair of electrodes. As a result, several natural and intuitive sensations of lost fingers were evoked by adjusting the amplitude, width, and frequency of the bidirectional rectangle wave when the stimulus was applied to the stump through electrode pairs, proving that the bidirectional rectangle wave composed of 10 pulses/s was the most effective stimulus mode. Approximately six types of sensations were observed, such as vibration, gentle touch, blunt pat, sharp pat, acmesthesia, and pressure. This phenomenon was particularly evident in six electrode pairs, which are marked with red arrows in Figure 4B, and the locations of the sensations that were most frequently evoked at each stimulus position in P1–P6 were, respectively, displayed with six different color blocks. Besides, these six positions were determined to be the proper stimulus positions (P1–P6) for inducing sensations in subject 1. Additionally, the same method based on PHM distribution was used in other subjects to choose optimal stimulus locations to evoke sensations. The results of the optimal stimulus positions varied among subjects due to their different stump conditions and PHM distributions. Table 2 lists the potentially effective stimulus parameters that were initially determined after the range test in Selections of stimulus waveforms and parameter ranges.

**Table 2:** Ranges of potential parameters that were pre-determined after the test experiment in section 2.2.2.

Frequency (Hz)	5, 20, 50, 75, 100, 150, 200
Wave width (μs)	100, 150, 200, 250, 300, 400
Amplitude (mA)	1.5, 2.0, 2.5, 3.0, 3.5, 4.0, 4.5, 5.0

Afterward, the stimulation parameters in Table 2 were tested on each determined stimulus position. The amplitude thresholds of the stimulus current at six determined stimulus positions (P1–P6) for subject 1 were recorded at a frequency of 50 Hz and a wave width of 200 μs in section 2.3. The threshold results included 2.0, 2.5, 3.0, and 4.0 mA (as shown in Supplementary Figure S2), and the frequency of each threshold was calculated by calculating the ratio of the number of experiments with the threshold to the total number of experiments. The results indicated that the amplitude thresholds of 2.5 and 3.0 mA have the highest probability of occurrence across all stimulation positions. Thus, a stimulus with an amplitude of 3.0 mA or greater can provide sufficient intensity to induce phantom finger sensations in most cases. Besides, three types of evoked sensations, including blunt pressure, vibration, and pressure, were frequently observed at stimulus frequencies of 5, 50, and 200 Hz, respectively. Furthermore, the different levels of intensity of evoked sensations were changed by adjusting the amplitudes and wave widths of stimulation currents. Therefore, a set of representative and distinct parameters were selected (listed in Table 3) and then used in the following long-term experiment to test the stability of the determined stimulation positions and parameters.

**Table 3:** Selected parameters tested in the long-term experiments.

**No**.	**1**	**2**	**3**	**4**	**5**	**6**	**7**	**8**
**Frequency (Hz)**	5			50				200
**Wave width (μs)**	200	200	300	100	200	200	300	200
**Amplitude (mA)**	3.0	5.0	5.0	5.0	3.0	5.0	5.0	5.0

**Figure 5:**
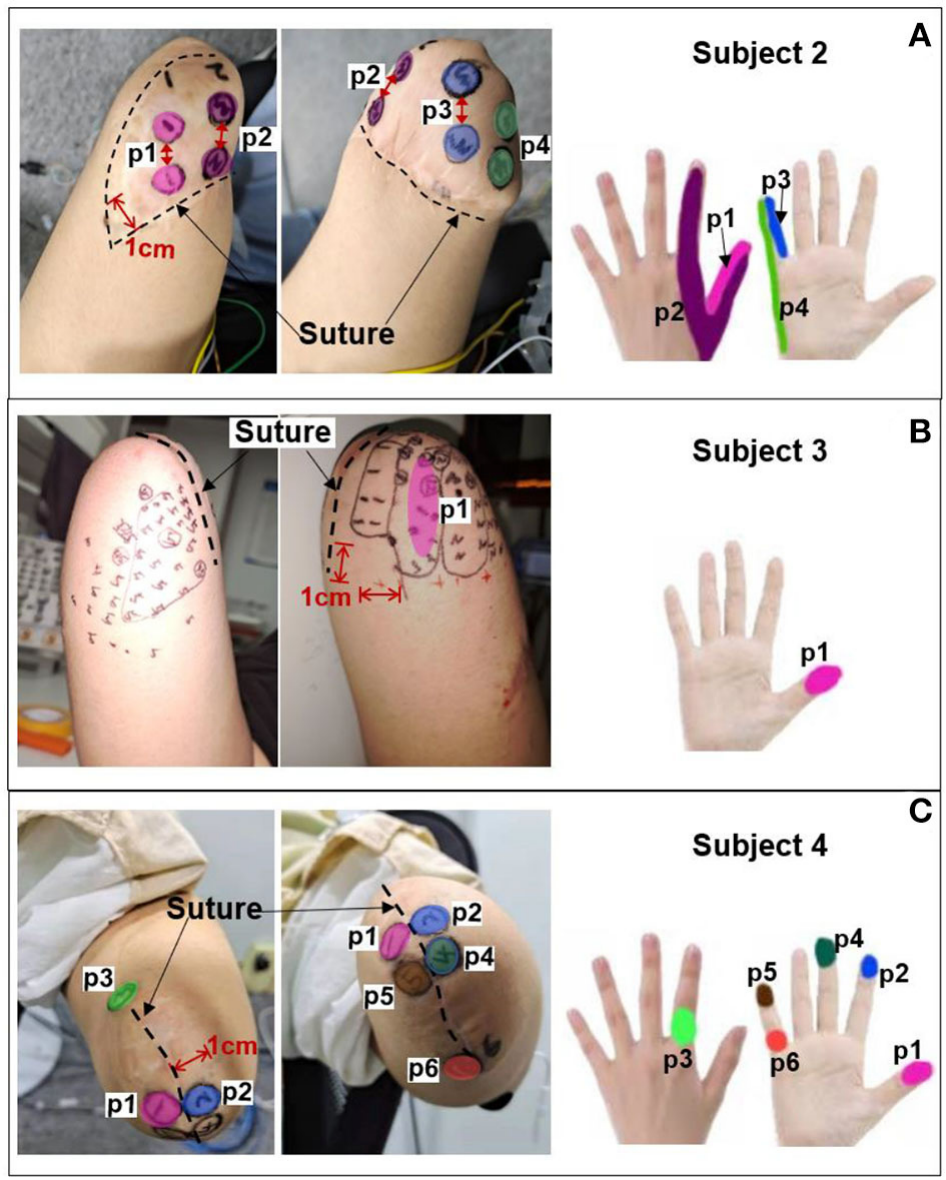
PHMs, determined stimulus positions, and corresponding sensation positions for additional three amputees. **(A)** Subject 2. **(B)** Subject 3. **(C)** Subject 4.

The PHMs on the stumps of the other three amputees (*subjects 2–4* in Table 1) were also examined to identify the appropriate electrical stimulation positions. Then, the effectiveness of the selected stimulus parameters in Table 3 was evaluated for their ability to elicit sensations in these amputees by applying TENS to the selected positions for each of them. It should be noted that the stimulation amplitude was adjusted from 0 to 10 mA. Figure 5 illustrates that the PHMs of the lost fingers are located around sutures on the stumps (indicated by black dotted lines), but their locations vary among different amputees. For instance, the PHMs of five lost fingers in subject 2 are located in areas between two sutures with a width of ~4 cm (for *subject 2*), while in subject 4, they are found in areas with a width of 1 cm (for *subject 4*). However, for *subject 3*, only PHMs of the thumb, index, and little fingers were found, which are located ~3–5 cm wide on both sides of the sutures. Afterward, some optimal positions at the stump were determined based on the distribution of PHMs for each amputee, and several stable sensations were evoked by applying TENS to these positions.

The corresponding relationship between the selected stimulus locations and the positions of sensations evoked by TENS for each subject is shown in Figures 5A–C. For *subjects 2* and *4*, sensations on five fingers could be elicited by TENS, but they were not consistent for all fingers despite the presence of PHMs on all fingers. Instead, stable sensations on the thumb, index, and little fingers for *subject 2* were evoked when TENS acted on the four determined stimulus positions p1–p4, as shown in Figure 5A. The position of each evoked sensation was in a relatively wide area on the finger, which was similar to the sensory positions of *subject 1*. For *subject 4*, stable sensations of the thumb, index, middle, and little fingers were induced when the skins at positions p1–p6 in Figure 5C were stimulated. Moreover, the subject was able to distinguish precise positions of the evoked sensations. For instance, sensations evoked at positions p1, p2, p3, and p4 were on the tips of the thumb, index, middle, and little fingers. Additionally, sensations induced by stimulation at p3 and p6 were located at the dorsal and ventral sides of the third knuckle of the index and little fingers, respectively. For *subject 3*, only the stable sensations on the tip of the thumb were evoked at one position (p1) by the TENS during the long-term experiment, as shown in Figure 5B. Therefore, the stable sensations of phantom fingers can be evoked by stimulating the appropriate positions, which were determined based on PHM distributions for all amputee subjects. The key point is that amputees had different PHMs, leading to different sensory positions in phantom hands induced by applying TENS to targeted stimulus locations. Furthermore, the sensitivity to stimulus amplitudes varied among subjects. For example, *subject 2* can perceive sensations from his phantom fingers when the amplitude changed from 3 to 5 mA. However, for *subjects 3* and *4*, sensations of their phantom fingers were evoked when the stimulus amplitude range was between 5–7 mA and 7–9 mA, respectively.

### 3.3. The effectiveness and stability of confirmed TENS configurations

**Figure 6:**
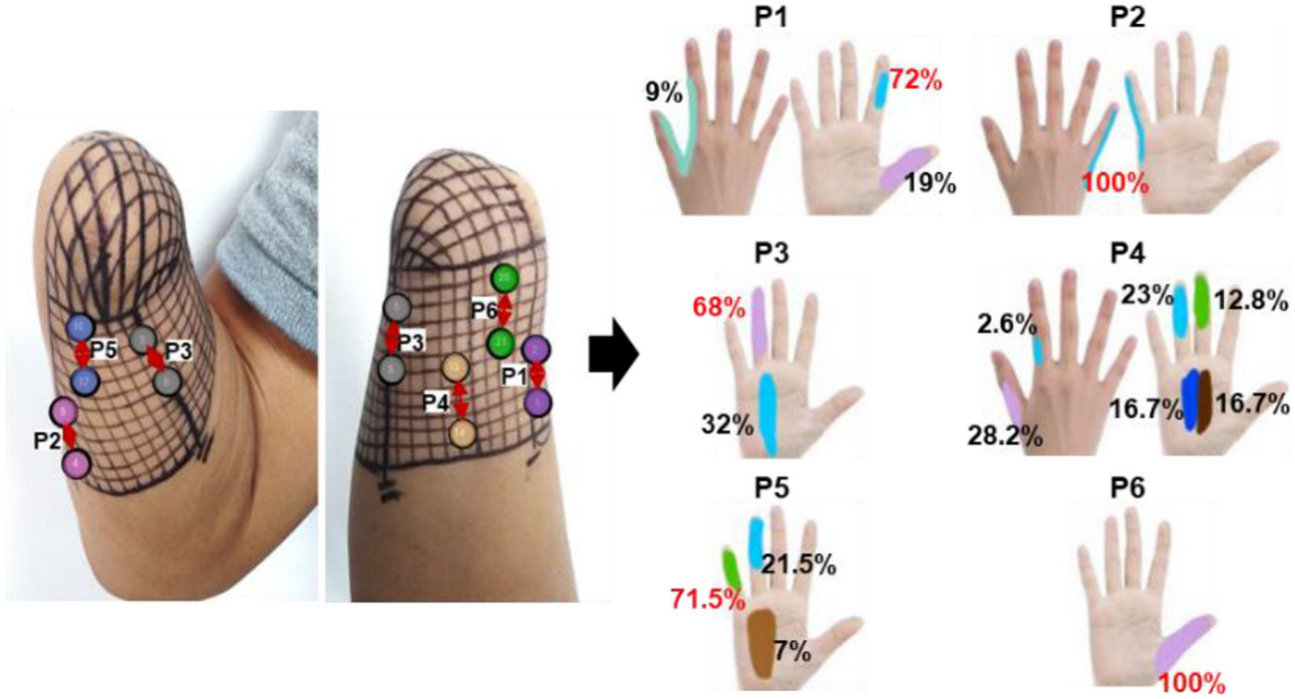
The positions of evoked sensations when the six selected positions were stimulated by TENS under the SS mode.

**Figure 7:**
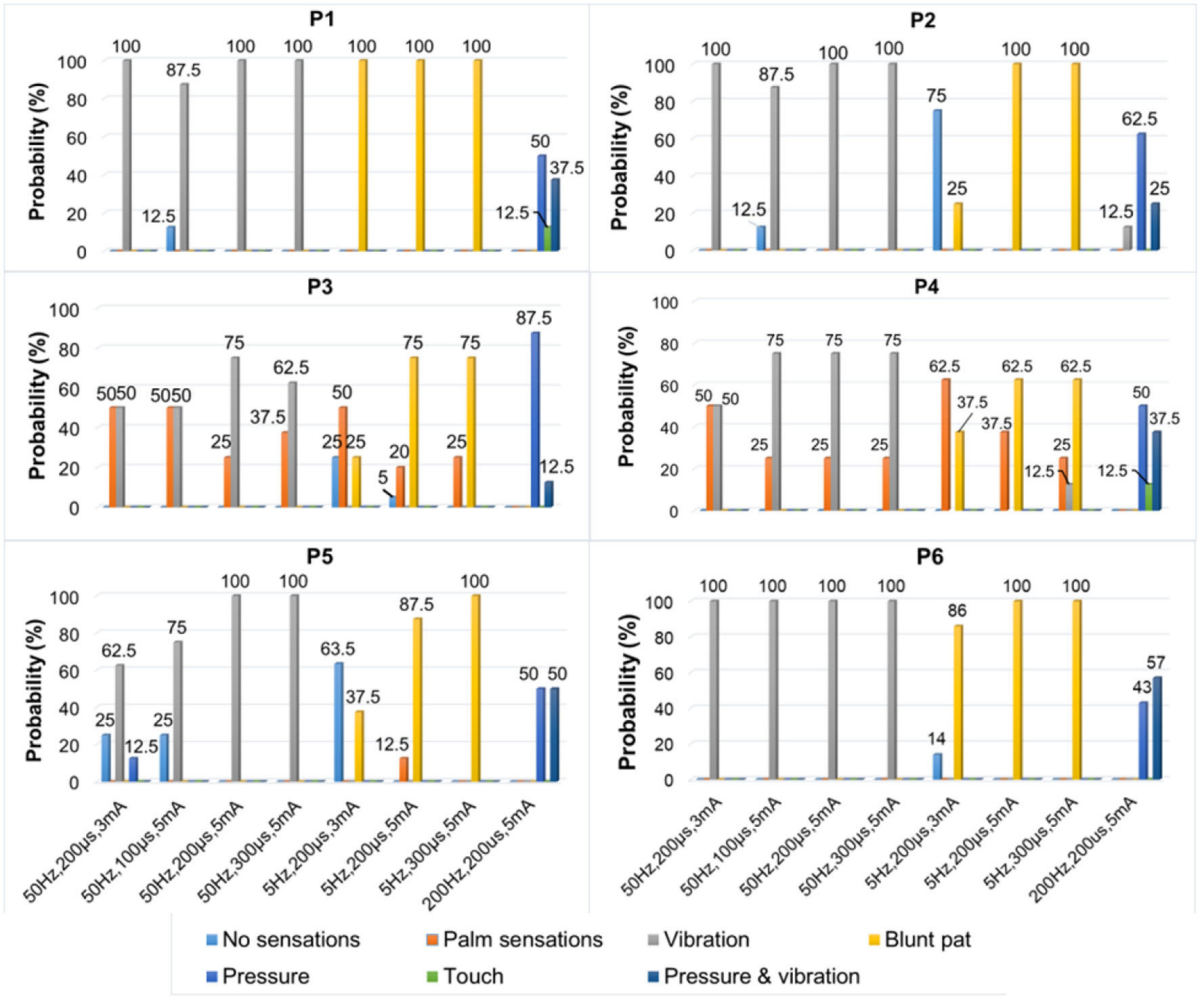
The types of evoked sensations when the six selected positions were stimulated by the TENS with selected parameters under the SS mode.

**Figure 8:**
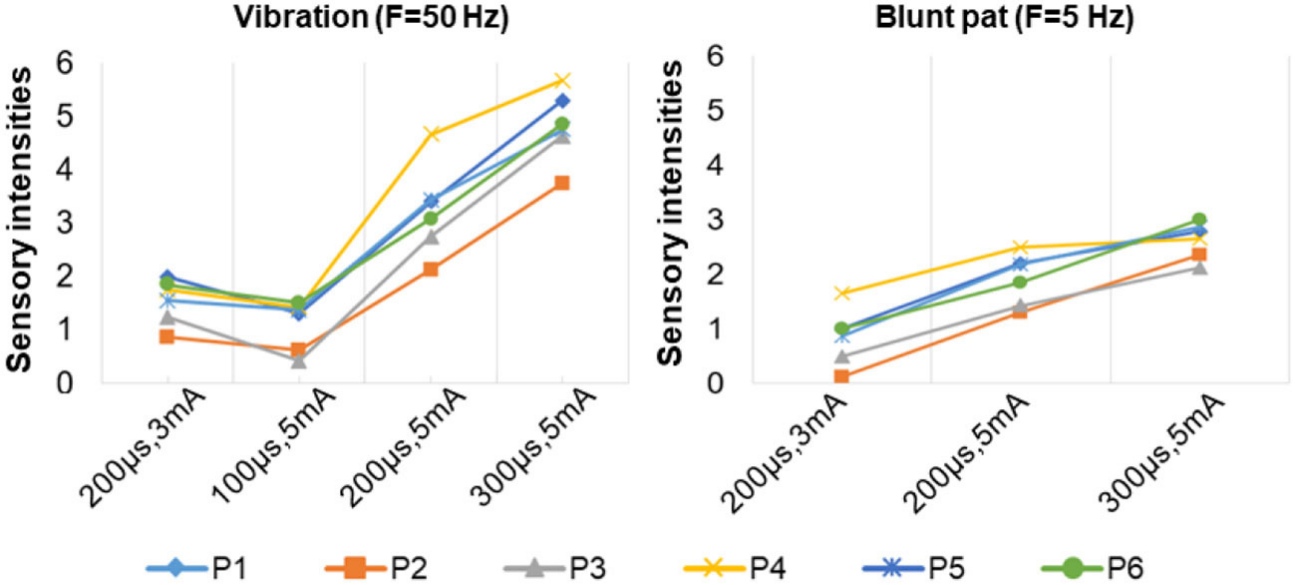
The relationship between sensation intensity and the stimulus parameters under the SS mode (the stimulus was 5 and 50 Hz).

In the long-term stability testing experiment, the positions, types, and different levels of intensity of the evoked sensations under each tested parameter were recorded. Then, the likelihood of each sensory parameter was determined by calculating the ratio of the number of experiments that induced the sensory parameter to the total number of experiments conducted under a specific test stimulus parameter. Figures 6–8 were all obtained from tests in the SS pattern for *subject 1*. Figure 6 shows the sensory positions induced at each of the six selected stimulus locations (P1-P6) and their frequency of occurrence. It indicates that the stable sensations of the thumb and little finger were consistently evoked and induced at P2 and P6 (with a frequency of 100%), and there was a high probability (81%) of inducing the index sensation at the P1 location. Furthermore, sensations of the middle and ring fingers, as well as the palm, can be evoked by applying TENS to stimulus positions of P3–P5, but the corresponding relationships of positions between evoked sensations and stimulations were not strictly stable. For instance, when the skin at P4 was stimulated, the occurrence probability of the elicited sensory position in the thumb, index finger, middle finger, ring finger, and palm was 28.2, 2.6, 12.8, 23, and 33.4%, respectively.

Figure 7 depicts a stable relationship between stimulus frequencies (5, 50, and 200 Hz) and sensation types (blunt pat, vibrations, and pressure). Especially for positions P1, P2, and P6, the probability of evoking the blunt pat and vibration sensations, respectively, under the stimulus frequencies of 5 and 50 Hz, was ~100%. However, there is a probability of 12.5% for P1 and P2, 75% for P2, and 14% for P6 of no sensation being evoked under the wave width of 100 μs and the stimulus amplitude of 3 mA. For positions P3–P5, the same matching relationship existed, but sometimes (P3–P4, 20–50% probability), the evoked sensation was the palm rather than the fingers. Moreover, when the wave width was 100 μs and the amplitude was 3 mA, P5 had no evoked sensation 25–63.5% of the time. In addition, when the stimulus frequency was 200 Hz, the probability of the pressure feeling and the mixed sensation of pressure and vibration for all stimulus positions was over 87.5%, and the probability of touch sensation induction in P1 and P5 was 12.5%. Figure 8 shows that the intensity of vibration and blunt pat sensations increases with an increase in stimulus amplitude and wave width at frequencies of 50 and 5 Hz for all stimulus positions.

**Figure 9:**
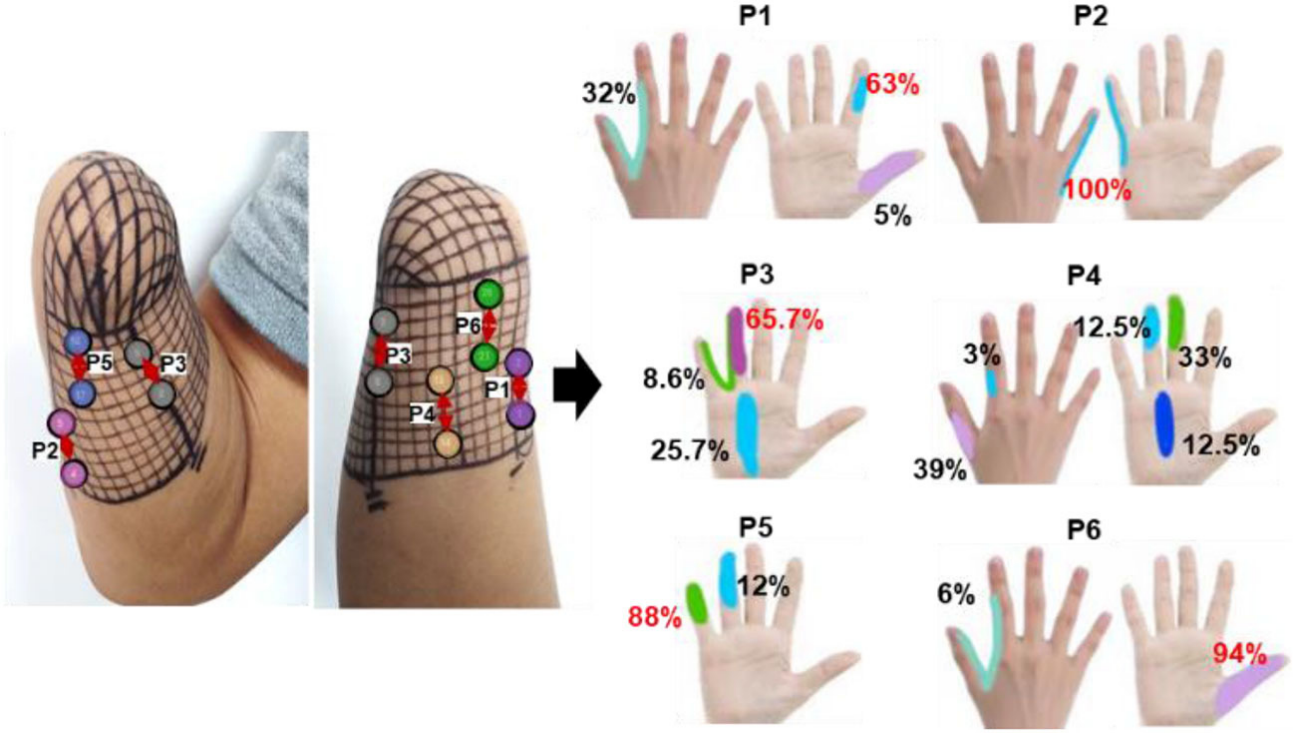
The positions of evoked sensations when the six selected positions were stimulated by TENS under the MS mode.

**Figure 10:**
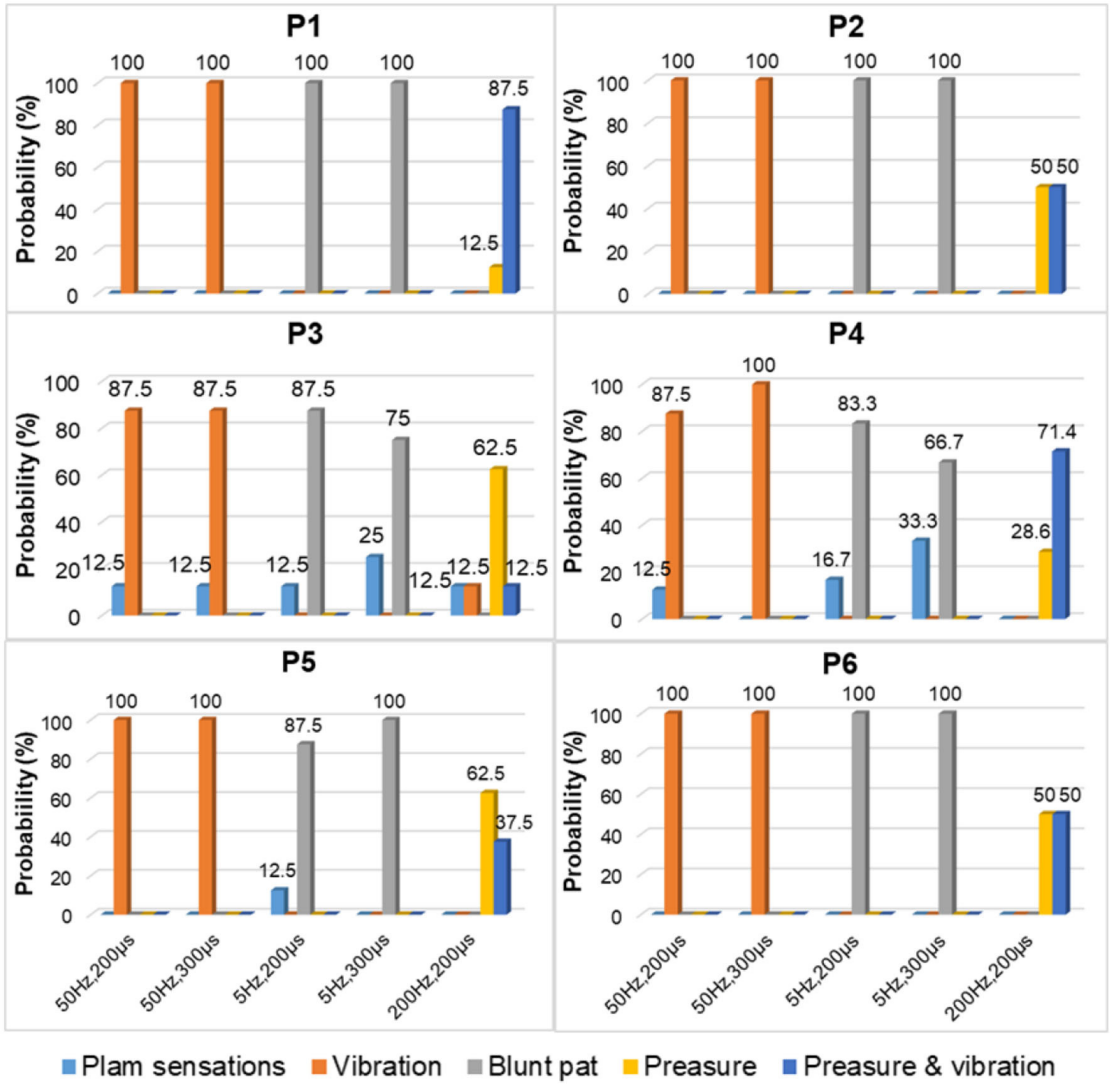
The TENS parameters and the types of evoked sensations on the six positions under the MS mode.

**Figure 11:**
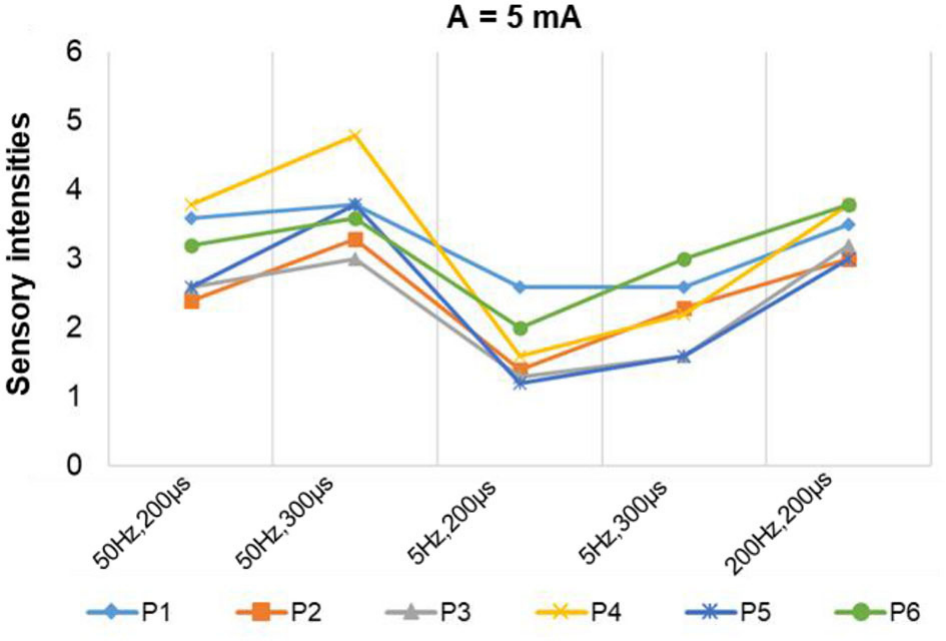
The stimulus parameters and the sensation intensities under the MS mode.

Figures 9–11 were all obtained from tests in the MS pattern for *subject 1*. Considering the subjects' tolerance for the duration of the experiment, parameters were filtered according to the result from the previous SS pattern test. The stimuli with an amplitude of 5 mA and a wave width >100 μs were further selected from Table 3 as they were dependable for evoking sensations. Figure 9 shows the corresponding relationship between stimulus positions and sensation positions. It is almost consistent with the result obtained in the SS pattern. The stable sensations of the thumb and little finger were evoked at P2 and P6 with a probability of 100%. A slight difference in the sensation of the index finger was also evoked on P6 at a small frequency of 6%. Moreover, the stability of the relationship between the positions of stimulation and evoked sensation is relatively improved under the MS pattern.

For instance, the relationship between P1 location and index finger sensation and P5 location and little finger sensation is up to 95 and 88%, respectively. At P4, the evoked sensations were still unstable in the MS pattern, but more sensations of the fingers than the palm were induced, compared with the result in the SS pattern (77.5 vs. 66.6%). Furthermore, the probability that the sensory positions were in the thumb, index finger, middle finger, ring finger, and palm was 39, 3, 33, 12.5, and 12.5%, respectively.

Figure 10 depicts the relationship between stimulus frequency and the type of sensation evoked in the MS pattern, with 5 Hz evoking a blunt pat sensation, 50 Hz evoking a vibration sensation, and 200 Hz evoking a pressure and a mix of pressure and vibration. Especially for stimulus positions P1, P2, and P6, the corresponding relationship is stable with a 100% probability. However, for stimulation locations of P3–P5, the stability was less satisfactory, as, sometimes, the evoked sensation was on the palm rather than on the fingers (12.5–33.3%). Figure 11 indicates that sensation intensity increases with the increasing stimulus wave width for almost all stimulus positions, but this changing relationship is not clear for P1 under a frequency of 5 Hz. Additionally, different levels of intensity of sensations evoked at 5 Hz were smaller compared to those evoked at 50 Hz and 200 Hz. However, under the stimulation wave width of 200 μs, the result showed no significant difference in the sensory intensity induced by TENS with frequencies of 50 and 200 Hz. Nevertheless, the match relationship between the selected stimulus configuration and evoked sensations was stable during a long-term experiment (especially for P2 and P6), either in the SS or the MS test pattern.

### 3.4. ERP of evoked sensations

**Figure 12:**
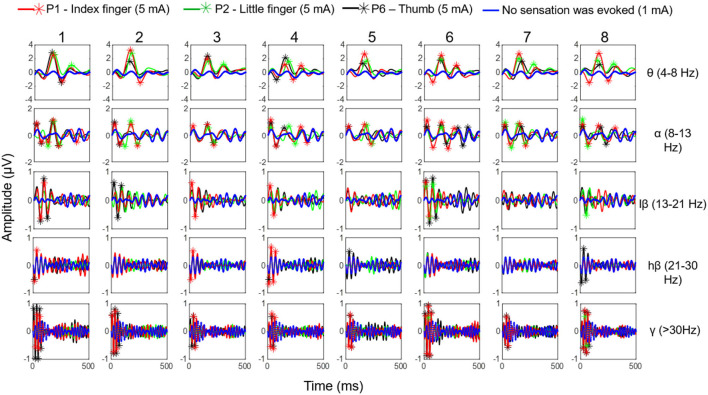
The ERP curves when stable sensations of the index finger, little finger, and thumb were evoked at stimulus positions P1, P2, and P6 under the amplitude of 5 mA as well as no sensation was evoked under the amplitude of 1 mA, in eight experiments (in MS pattern) for *subject 1*.

According to the stability result of the match relationship among stimulation parameters, positions, and evoked sensations obtained from section 3.3, the EEG signals corresponded to stable pressure sensations in the index, little, and thumb fingers, which were evoked by TENS at stimulus locations P1, P2, and P6. The signals were recorded during the long-term test in the MS pattern under the stimulation parameters of 5 mA, 200 Hz, and 200 μs. The average ERPs of selected channels for five EEG rhythms (θ, α, lβ, hβ, and γ) were calculated. Figure 12 demonstrates the ERP curves when stable sensations in the index finger, little finger, and thumb were evoked at stimulus positions P1, P2, and P6 under the amplitude of 5 mA, as well as no sensation was evoked under the amplitude of 1 mA, in eight experiments for *subject 1*. It illustrates that once the sensations were successfully induced, the ERPs peaks appeared, and the corresponding band of ERPs frequently focused on θ, α, and lβ rhythms, i.e., 4–21 Hz. For sensations in the thumb and index finger, their corresponding ERPs also included some components in the γ frequency band. Moreover, in θ and α bands, the ERP peaks of the little finger were generated later than the thumb and index finger, and the peak values of the thumb were higher than those of the index and little fingers in most cases, reaching over 2 μV.

## 4. Discussions

The recovery of natural and intuitive phantom hand sensations is crucial for amputees. However, existing commercial solutions fail to meet this demand. Although invasive methods can induce sensations similar to those perceived in intact limbs, identifying the precise nerve fibers remains a challenge. Furthermore, challenges such as biological compatibility, power supply, heat, and long-term stability remained unresolved, restricting the implantation of microstimulation systems and limiting the use of invasive methods. Therefore, effective and non-invasive technology is desired by amputees, investigators, and clinicians. One example of this is TENS, which has the potential to induce somatotopic and homologous sensations by stimulating the skin surface of amputees' stumps (Raspopovic et al., 2021). However, TENS is not without its flaws and can be difficult to administer in a clinical setting due to challenges in identifying precise stimulation points and determining optimal stimulation settings. Despite these drawbacks, research has yielded promising results, highlighting the significance of PHM in eliciting intuitive sensations of phantom fingers.

Studies have shown that PHMs are found in the stumps of some amputees, and their different distributions vary among different amputees. Thus, we have explored the distribution law of the PHMs for amputee subjects by using mechanical stimulation in a long-term experiment in this study, which can be useful in confirming stimulus positions in the TENS experiments. Previous studies suggested that using a grid coordinate on the stump is the most effective method to ensure the accuracy of each test position and its consistency in cross-day experiments. It should be noted that the subject consented to have the grid remain on his stump until the end of the study (as shown in Figure 1). The results show that the distribution of PHM was not identical across different days of experimentation, as the participant reported experiencing variations in phantom finger sensations when the same grid coordinates were stimulated on different days. One possible reason for the difference may be that the intensity and direction on different days of the pressure acting on the same mesh were not consistent, which were not at even different meshes on the same experiment day. On the other hand, the arm posture, the state of the skin, and the physical situation of the subject may also cause differences in results. Nevertheless, results in section 3.1 demonstrate that the PHMs' distribution is still regular, notable for many meshes. The sensations of several phantom fingers with different levels of intensity may be simultaneously induced on one grid. This also possibly causes differences in PHMs tests, as it may indicate the regeneration of several nerve fibers, each with different sensitivity to stimuli for different fingers, at the same grid position in the stump. For *subject 1*, PHMs covered areas with a length of ≈ 10 cm, which is over half the length (≈ 15 cm) of the stump with a width of ≈ 12 cm. Figure 3 shows that PHMs are present on his five fingers. Therein, the PHMs of the thumb and little finger are mainly distributed on the ulnar and redial sides, respectively. Additionally, PHMs for the mixture of the thumb and index finger, as well as the ring and little fingers, are almost evenly distributed with those of the thumb and little finger, respectively. This could demonstrate the abundant regeneration of ulnar and redial nerve branches around the severed main nerves in the stump for *subject 1*. Furthermore, the subsequent TENS experiments also showed that it is easier to induce the sensations for the phantom thumb and little finger than for other phantom fingers in terms of selecting appropriate stimulus positions. The distribution laws of PHMs were then used to explore effective TENS configurations for sensation induction.

The results of screening and verifying appropriate stimulation positions and effective stimulus parameters have been described in section 3.2. Nineteen stimulus locations were preliminarily selected to test the ability of TENS on sensation inductions according to the distributions of PHMs, as shown in Figure 4A. The sensations in each position are clearer than others and are associated with one specific phantom finger. However, the sensory positions evoked by TENS are somewhat different from those evoked by mechanical stimulation, which may be a result of the bigger size of the electrode (1 cm) used in electrical stimulation, the wider action range under the skin of the electric field, and the different neural activation mechanisms between the two methods. Nevertheless, the PHM distribution obtained through mechanical stimulation is still helpful for finding proper stimulus locations in TENS, probably due to the accomplishment of the nerve fibers' regeneration or the stabilization of the brain's functional organization after a long time since the amputation (≈ 14 years so far for subject 1). Finally, six suitable stimulation locations (P1–P6 for subject 1), as shown in Figure 4B, were confirmed, and then, the relative stability of the relationship between the stimulus locations and the evoked sensory positions was verified through long-term SS and MS experiments (Figures 6, 9). Especially at positions P1, P2, and P6, sensations of the phantom index finger, the little finger, and thumb can be, respectively, induced with high probabilities by TENS, even 100% for the little finger at P2, in both single and continuous stimulation experiments. P1, P2, and P6 are suitably located at PHMs for the mixture of the thumb and index finger on the ulnar side, as well as at PHMs for the little finger on the radial side. This could also demonstrate the abundant regeneration of ulnar and radial nerve branches in these three regions, which may be a reason for stabilizing evoked sensations on the above three phantom fingers. Furthermore, sensations of both the thumb and index finger were induced at P1 and P6 but with different possibilities, which may indicate that the nerve fibers of the two fingers have different types, different levels of intensity, and different levels of sensitivity to stimuli at P1 and P6, and they may come from different general nerve branches but may also come most likely from the ulnar nerve. Besides, feelings in the ring and little fingers, and hypothenar palms can be induced with different possibilities by stimulating positions P3 and P5, showing that fibers from the radial nerve branches may exist in these areas. The sensations in the thumb, middle finger, ring finger, and center palm could be evoked at some times when TENS was applied to P4. This may demonstrate the existence in the P4 area of fibers from the median nerve branches. It is worth noting that the other five of the six confirmed positions are PHMs that have mixed finger sensations, except for P2. Moreover, at each of these five locations, the possibilities for evoking sensations with the mixed phantom fingers have more or fewer differences between the SS and MS experiments, as shown in Figures 6, 9. This could prove that continuous stimulation has different effects on the sensitivity and adaptability of different nerve fibers to the stimulation.

For the stimulus parameters, the bidirectional rectangle wave composed of 10 pulses/s was the optimal stimulus pulse for eliciting various sensations by changing the amplitudes, frequencies, and wave widths in this study. Additionally, the evoked sensations were reported to be more comfortable with these pulses than with other types of pulses, which was reported by the subject and could be owed to the discreteness of the stimuli pulses. The amplitude thresholds vary at the six selected positions but are nearly within the range of 2.5–3.0 mA, indicating different levels of intensity, varying degrees of sensitivity to stimuli, and firing mechanisms of different fibers in these areas. Notably, these thresholds were only for *subject 1*; they could be different among other amputees due to different stump situations and PHMs, but the testing method can be applied to others. Moreover, various types of sensations were successfully induced, and most of them were pleasant, such as touch, a blunt pat, vibrations, and pressure. Figures 7, 10 shows that different types of sensations were evoked by adjusting the frequencies of stimuli, including 5, 50, and 200 Hz, and these results were consistent across the six selected stimulus positions. This may prove that the stimulus current bypassed receptors in the stump skin at the target PHMs and directly activated the nerve fibers in these areas; otherwise, the above results would not be consistent for different stimulus positions. One explanation could be that different types of receptors respond differently to the same stimulation parameter, and their distribution at a specific stimulus position is heterogeneous. Additionally, some unpleasant sensations like pain, itching, and tingling also could be evoked in some cases, such as under the wave-width >350 μs, but these kinds of unpleasant sensations are also useful since they are common feelings in daily life. The subject reported no discomfort other than the evoked sensations. Furthermore, one of the main purposes of this study is to confirm a set of effective and stable TENS parameters for the stable induction of phantom finger sensations. The selected parameters are listed in Table 3. The results of the relationship between sensory types and stimulus parameters in Figures 7, 10 indicate that three kinds of stable sensations, including blunt touch, vibration, and pressure, were evoked at frequencies of 5, 50, and 200 Hz, respectively. This stable correspondence is clear at positions P1, P2, and P6, which is consistent in SS and MS pattern tests. This may be because nerve fibers are more concentrated and dense in these areas than in other locations, proving that positions P1, P2, and P6 are the optimal stimulus positions to induce stable phantom finger sensations. Besides, the stability of the corresponding relationship between selected positions in the MS pattern is stronger than those in the SS pattern, which may be due to the adaptation and fatigue of nerve fibers to stimuli. Therefore, future studies on practical applications should consider appropriate stimulus intervals. Figures 8, 11 prove that the intensity of evoked sensations could be strengthened or weakened by the increase or decrease of stimulus amplitudes or wave widths because the energy injected into the target stimulus location could be changed by changing the amplitude or pulse width of the current, which may lead to different effects on the firing of nerve fibers.

The PHMs vary among different amputees in terms of distribution ranges, locations, and corresponding parts of phantom hands. Therefore, three other subjects with different amputation conditions (*subjects 2-4* as shown in Table 1) were included in the long-term experiment to explore the PHM distributions on their stumps and to verify the effectiveness and robustness of TENS parameters. The results show that sensations of phantom fingers were successfully induced only when TENS was applied to appropriate PHMs. This was consistent for all subjects, despite their having different PHM distributions and stump states. Additionally, different finger sensations corresponded to different stimuli positions. Once the optimal stimulus location was determined, the position of the evoked sensation was also confirmed. In this situation, the sensation types only depend on the stimulus parameters rather than being influenced by the stimulation locations. Additionally, the stimulus parameters confirmed in section 3.2 were suitable for other amputees because subjects reported the same sensation types when their target PHMs were stimulated by the electrical pulses with the same parameters as *subject 1*. However, the sensation intensity under the same parameters and the parameter thresholds of effective stimulus pulses varied among different subjects, which may be due to various amputations, stumps, or physical conditions, and nerve regenerations and reorganizations after their amputations. Both the condition and the length of the stump could strongly influence the quality and distribution of PHM, which could influence the TENS for sensory feedback. Prior research showed that the longer the stump, the more detailed the representation of fingers could be in the PHM, and the clearer phantom finger sensations could be induced with TENS (Chai et al., 2015). This result was not clear for the four subjects with wrist joint level amputations in our study; however, we found that factors such as the degree of atrophy in stump muscles and the cause of amputation, such as trauma or tumors, also influence the quality of PHM and the efficiency of TENS-evoked sensations. For instance, *subject 1*, who was amputated (stump length ≈ 15 cm) due to mechanical trauma, had large-size PHMs of five lost fingers on the stump and was able to experience sensations of all fingers when TENS was applied, but the probabilities and stability will only be for three fingers. Similarly, *subject 2*, who was also amputated due to trauma (stump length ≈ 15 cm), had PHMs on five fingers, but they only covered a small part of the residual limb. Besides, stable sensations of only three fingers (thumb, index finger, and little finger) could be evoked by TENS during the long-term experiments, as shown in Figure 5A. For *subject 3*, the mechanical trauma and high-temperature burn led to his amputation. Despite possessing a long residual limb (stump length ≈ 25 cm) and clear PHMs of some fingers including the thumb, index, and little fingers, TENS only evoked his thumb sensation, as shown in Figure 5B. Besides, he occasionally suffered from severe phantom limb pain, and thus any touch and electrical stimulus on his skin would cause intolerable pain, which prevented the sensation-evoking. Unlike the others, *subject 4* (stump length ≈ 14 cm) underwent a wrist amputation of his own due to a complex tumor on his terminal limbs, and his stump had a regular surgical incision with an orderly shape, as shown in Figure 5C. Besides, the PHMs of five fingers were easily found, although the size of his PHMs is the smallest one among the four subjects, and the sensations of all fingers even on the fingertips could be stably evoked by TENS. Therefore, the confirmed parameters are applicable for all subjects in this work, but the customization of stimulus positions is needed for different amputees with different stump conditions.

ERPs results for the phantom thumb, index finger, and little finger sensations evoked at the optimal positions P1, P2, and P6 for *subject 1* are displayed in Figure 12. This figure shows that ERP peaks appear only when sensations are successfully induced, proving objective evidence of the effectiveness of TENS in sensation induction. Differences in the main frequency bands of ERPs and amplitude values of ERP peaks among the three fingers may indicate differences in brain activity in response to the evoked sensations of different fingers. This could also provide evidence for the validity of the confirmed stimulus parameters in this study. However, the ERP curves of the thumb and the index finger are relatively smaller, which could be attributed to the close distance between the stimuli electrodes at P1 and P2. This may indicate that some of the same neurons and nerve bundles are active when stimulation is applied to P1 or P2, resulting in interference with EEG signals. In future studies, it would be ideal to obtain potential brain activity independently. In future studies, it would be ideal to independently measure potential brain activity. However, currently, it is challenging to do so due to the complex mixture of signals coming from various sources in the brain caused by field spread and volume conduction (He et al., 2019; Ding et al., 2020). Further research is needed to improve these techniques and better understand the underlying mechanisms.

Our experimental process had several limitations, despite using innovative and original methods. On the one hand, the process of determining the locations of PHMs with a mesh coordinate on the arm was difficult for some subjects to accept. Therefore, we should look for better methods to precisely mark the locations of PHMs in future studies, such as customizing wearable devices for subjects instead of drawing coordinates. On the other hand, during body motions with different gestures, the relative position of the nerves concerning the electrodes may change, which would influence the activation charge and, thus, the perceived sensation (Svensson et al., 2017). In this study, we found that changes in arm posture could result in differences in evoked sensations when the TENS was applied to some locations of the stump. When it came to the specifically selected positions in this study, however, the aforementioned differences were negligible. In the experiments across different days, the subject was electrically stimulated without a fixed posture, but consistent sensations were evoked. Therefore, the selection of the optimal stimulus positions is pivotal for stable sensation induction. Additionally, the skin condition is also one of the critical factors that affect the stimulation intensity. Since some objective parameters, such as skin moisture and temperature, were not constant during the experiments, the conductivity of the electrode was also changeable (Raspopovic et al., 2021). In future studies, we may use a combination of invasive and non-invasive techniques to investigate more stable and efficient methods for sensation induction. We are also exploring the use of microneedle electrodes for more accurate stimulation of nerve endings in the active epidermis, which may help overcome the problem caused by the relative movement of the electrode position.

## 5. Conclusion

In this study, an optimal TENS strategy was developed to induce natural and intuitive phantom hand sensations for amputees. The effectiveness and stability of the confirmed stimulus configurations were evaluated through different stimulation modes during a long-term follow-up experiment. Additionally, EEG signals throughout the brain were recorded while the TENS with confirmed parameters was applied to the selected positions. Then, the evoked sensations were also assessed by calculating ERPs. In this study, different kinds of sensations were induced by adjusting the frequencies of stimulus pulses. Besides, it was found that the intensity of the sensation was strongly related to the stimulus amplitudes and wave widths. The performance of the selected parameters was found to be stable in terms of sensory induction, and these results were consistent for different amputee subjects. Additionally, the various stump conditions resulted in different distributions of phantom hand movements (PHMs) among amputees, leading to different effects of TENS on sensory inductions. This result could guide us in customizing stimulus configurations for different amputees according to their PHMs, especially for the selection of stimulation locations. In future studies, we plan to recruit more amputee subjects to participate in the experiment to explore statistically significant consistency rules among different patients and to analyze EEG signals from various amputees to search for the mechanisms underlying brain activities. Additionally, we will attempt to relate the application of the TENS strategy developed in this study to the treatment of other diseases.

## Data availability statement

The raw data supporting the conclusions of this article will be made available by the authors, without undue reservation.

## Ethics statement

The research protocol was approved by the Institutional Review Board of Shenzhen Institutes of Advanced Technology, Chinese Academy of Sciences (IRB Number: SIAT-IRB-190315-H0325). The patients/participants provided their written informed consent to participate in this study.

## Author contributions

Conceptualization: YW, PZ, and YT. Data curation: YW, HL, and LJ. Methodology, validation, and visualization: YW. Funding acquisition: YT. Writing: YW, YZ, LZ, and YT. All authors contributed to the article and approved the submitted version.

## References

[B1] Antfolk C. D'Alonzo M. Controzzi M. Lundborg G. Rosen B. Sebelius F.. (2013). Artificial redirection of sensation from prosthetic fingers to the phantom hand map on transradial amputees: vibrotactile versus mechanotactile sensory feedback. IEEE TNSRE. 21, 112–120. 10.1109/TNSRE.2012.2217989 23033439

[B2] Björkman A. Weibull A. Olsrud J. Ehrsson H. H. Rosén B. Björkman-Burtscher I. M. (2012). Phantom digit somatotopy: a functional magnetic resonance imaging study in forearm amputees. Eur. J. Neurosci. 36, 11–19. 10.1111/j.1460-9568.2012.08099.x 22537316

[B3] Björkman A. Wijk U. Antfolk C. Björkman-Burtscher I. Rosén B. (2016). Sensory qualities of the phantom hand map in the residual forearm of amputees. J. Rehabil. Med. 48, 365–370. 10.2340/16501977-2074 26999267

[B4] Chai G. Sui X. Li S. He L. Lan N. (2015). Characterization of evoked tactile sensation in forearm amputees with transcutaneous electrical nerve stimulation. J. Neural. Eng. 12, 066002. 10.1088/1741-2560/12/6/066002 26401550

[B5] D'Anna E. Petrini F. M. Artoni F. Popovic I. Simanic I. Raspopovic S.. (2017). A somatotopic bidirectional hand prosthesis with transcutaneous electrical nerve stimulation based sensory feedback. Sci. Rep. 7, 10930. 10.1038/s41598-017-11306-w 28883640 PMC5589952

[B6] Ding K. Dragomir A. Bose R. Osborn L. E. Seet M. S. Bezerianos A.. (2020). Towards machine to brain interfaces: sensory stimulation enhances sensorimotor dynamic functional connectivity in upper limb amputees. J. Neural Eng. 17, 035002. 10.1088/1741-2552/ab882d 32272463

[B7] Ehrsson H. H. Rosén B. Stockselius A. Ragn,ö C. Köhler P. Lundborg G.. (2009). Upper limb amputees can be induced to experience a rubber hand as their own. Brain 131, 3443–3452. 10.1093/brain/awn297 19074189 PMC2639202

[B8] Flesher S. Collinger J. Gaunt R. (2016). Intracortical microstimulation of human somatosensory cortex. Sci. Transl. Med. 8, 361ra.141. 10.1126/scitranslmed.aaf8083 27738096

[B9] Flesher S. Downey J. Bensmaia S.. (2017). “Intracortical microstimulation as a feedback source for brain-computer interface users,” in Brain-Computer Interface Research: A State-of-the-Art Summary 6(M) (Cham: Springer), 43–54.

[B10] Granata G Iorio R. D. Romanello R. Iodice F. Raspopovic S. Petrini F.. (2018). Phantom somatosensory evoked potentials following selective intraneural electrical stimulation in two amputees. Clin. Neuro. 129, 1117–1120. 10.1016/j.clinph.2018.02.138 29625342

[B11] Gu G. Zhang N. Xu H. Lin S. Yu Y. Chai G.. (2021). *A soft neuroprosthetic hand providing simultaneous myoelectric control and tactile feedback*. Nat. Biomed. Eng. 16, 1–0. 10.1038/s41551-021-00767-0 34400808

[B12] Hao M. Chou C-H. Zhang J. Yang F. Cao C. Yin P.. (2020). Restoring Finger-Specific Sensory Feedback for Transradial Amputees via Non-Invasive Evoked Tactile Sensation. IEEE Open J. Eng. Med. Biol. 1, 98–107. 10.1109/OJEMB.2020.2981566 35402945 PMC8979634

[B13] He B. Astolfi L. Valdés-Sosa P. A. Marinazzo D. Palva S. O. Bénar C-G.. (2019). Electrophysiological Brain Connectivity: Theory and Implementation. IEEE Transac. Biomed. Eng. 7, 1–1. 10.1109/TBME.2019.2913928 31071012 PMC6834897

[B14] Kuiken T. A. Li G. Lock B. A. Lipschutz R. D. Mille L. A. Stubblefield K. A.. (2009). Targeted muscle reinnervation for real-time myoelectric control of multifunction artifcial arms. JAMA. 301, 619–628. 10.1001/jama.2009.116 19211469 PMC3036162

[B15] Kuiken T. A. Marasco P. D. Lock B. A. Harden R. N. Dewald J. P. A. (2007b). Redirection of cutaneous sensation from the hand to the chest skin of human amputees with targeted reinnervation. Proc. Natl. Acad. Sci. U. S. A. 104, 20061–20066. 10.1073/pnas.0706525104 18048339 PMC2148422

[B16] Kuiken T. A. Miller L. A. Lipschutz R. D. Lock B. A. Stubblefield K. Marasco P. D.. (2007a). Targeted reinnervation for enhanced prosthetic arm function in a woman with a proximal amputation: a case study. Lancet. 369, 371–380. 10.1016/S0140-6736(07)60193-7 17276777

[B17] Li K. Fang Y. Zhou Y. Liu H. (2017). Non-invasive stimulation-based tactile sensation for upper-extremity prosthesis: a review. IEEE Sens. J. 17, 2625–2635. 10.1109/JSEN.2017.2674965

[B18] Makin T. R. de Vignemont F. Faisal A. A. (2017). Neurocognitive barriers to the embodiment of technology. Nat. Biomed. Eng. 1, 1–3. 10.1038/s41551-016-0014

[B19] Osborn L. E. Betthauser J. L. Hunt C. L. Nguyen H. H. Kaliki R. R. Thakor N. V.. (2018). Prosthesis with neuromorphic multilayered e-dermis perceives touch and pain. Sci. Robot. 3, eaat3818. 10.1126/scirobotics.aat3818 32123782 PMC7051004

[B20] Overstreet C. K. Cheng J. Keefer E. (2019). Fascicle specifc targeting for selective peripheral nerve stimulation. J. Neural Eng. 16:066040. 10.1088/1741-2552/ab4370 31509815

[B21] Raspopovic S. Valle G. Petrini F. M. (2021). Sensory feedback for limb prostheses in amputees. Nat. Mater. 20, 925–939. 10.1038/s41563-021-00966-9 33859381

[B22] Schmalzl L. Thomke E. Ragnö C, Nilseryd, M. Stockselius A. Ehrsson H. H. (2011). Pulling Telescoped Phantoms Out of the Stump: Manipulating the Perceived Position of Phantom Limbs Using a Full-Body Illusion. Front. Human Neurosci. 5, 121. 10.3389/fnhum.2011.00121 22065956 PMC3206583

[B23] Shin H. Watkins Z. Huang H. Zhu Y. Hu X. (2018). Evoked Haptic Sensations in the Hand via Non-Invasive Proximal Nerve Stimulation. J. Neural Eng. 15, 046005.1-046005.12. 10.1088/1741-2552/aabd5d 29638220

[B24] Strauss I. Valle G. Artoni F. D'Anna E. Granata G. Iorio R. D.. (2019). Characterization of multi-channel intraneural stimulation in transradial amputees. Sci. Rep. 9, 19258. 10.1038/s41598-019-55591-z 31848384 PMC6917705

[B25] Svensson P. (2017). A review of invasive and non-invasive sensory feedback in upper 645 limb prostheses. Exp. Rev. Med. Dev. 14, 439–47. 10.1080/17434440.2017.1332989 28532184

[B26] Valle G. Mazzoni A. Iberite F. D'Anna E. Strauss I. Granata G.. (2018). Biomimetic intraneural sensory feedback enhances sensation naturalness, tactile sensitivity, and manual dexterity in a bidirectional prosthesis. Neuron. 100, 1–9. 10.1016/j.neuron.2018.08.033 30244887

[B27] Wang Y. Fang P. Tang X. (2022). Effective evaluation of finger sensation evoking by non-invasive stimulation for sensory function recovery in transradial amputees. IEEE Transac. Neural Syst. Rehabil. Eng. 30, 519–528. 10.1109/TNSRE.2022.3155756 35235514

[B28] Zollo L. Di Pino G. Ciancio A. L. Ranieri F. Cordella F. Gentile C.. (2019). Restoring tactile sensations via neural interfaces for real-time force-and-slippage closed-loop control of bionic hands. Sci. Robot. 4. *eaau*9924. 10.1126/scirobotics.aau9924 31620665 PMC6795534

