# Supplementary material for: Electrical nerve stimulation for sensory-neural pathway reconstruction in upper-limb amputees
Source: Front Neurosci. 2023 Feb 9;17:1114962. doi: 10.3389/fnins.2023.1114962 (PMC9947467; doi:10.3389/fnins.2023.1114962)
Supplement: Supplementary file 1 [file Data_Sheet_1.docx]

Supplementary Material

An Electrical Nerve Stimulation Strategy to Reconstruct SensoryNeural Pathways between the Stump and Brain for Amputees

In the result section of **3.1**, the total number of cells in eight experiments, which represent the PHM areas for each finger, were counted to measure the size of PHMs for different kinds of fingers. **Figure S1** demonstrates that the size PHM for the little finger is the largest (≈ 90), followed by the mixture of the ring and little fingers (≈ 80). Furthermore, PHMs for the thumb, index, and ring fingers, respectively had similar numbers of occurrences in the grid (≈ 20) with PHMs for three kinds of mixed fingers: thumb and index finger (I+II), middle and ring fingers (III+IV), as well as index, ringer, and little fingers (II+IV+V).

**Figure S1.** The number of sensory afferent locations (PHMs) for single or mixed fingers during eight experiments (represented by eight colored lines).

In the result section **3.2** of TENS parameter selection, the amplitude thresholds at six optimal stimulation positions are demonstrated in the **figure S2**, it shows the amplitude thresholds including 2.0, 2.5, 3.0, and 4.0 mA. And the appearing frequency of each threshold in eight repetitive experiments were calculated by the ratio that the number of experiments with the threshold to the total number of experiments. It indicated that the amplitude thresholds of 2.5 and 3.0 mA have the maximum probability of occurrence for all stimulation positions.


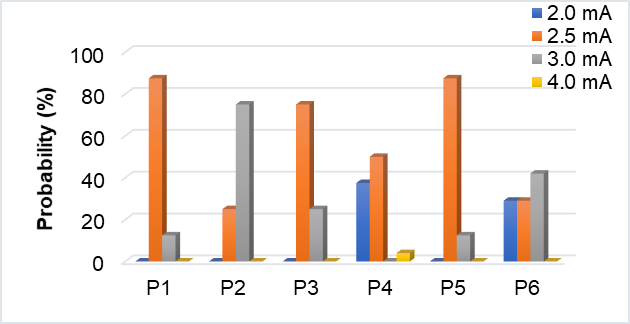


**Figure S2.** The stimulus amplitude thresholds were tested at determined positions of P1-P6 for *subject 1*.
